# Human Metabolic Network: Reconstruction, Simulation, and Applications in Systems Biology

**DOI:** 10.3390/metabo2010242

**Published:** 2012-03-02

**Authors:** Ming Wu, Christina Chan

**Affiliations:** 1 Department of Computer Science and Engineering, Michigan State University, East Lansing, MI 48824, USA; 2 Department of Chemical Engineering and Material Scince, Michigan State University, East Lansing, MI 48824, USA; 3 Department of Biochemistry and Molecular Biology, Michigan State University, East Lansing, MI 48824,USA

**Keywords:** human metabolic network, reconstructing context-dependent metabolic network, Constraint Based Modeling, simulation, systems biology

## Abstract

Metabolism is crucial to cell growth and proliferation. Deficiency or alterations in metabolic functions are known to be involved in many human diseases. Therefore, understanding the human metabolic system is important for the study and treatment of complex diseases. Current reconstructions of the global human metabolic network provide a computational platform to integrate genome-scale information on metabolism. The platform enables a systematic study of the regulation and is applicable to a wide variety of cases, wherein one could rely on *in silico* perturbations to predict novel targets, interpret systemic effects, and identify alterations in the metabolic states to better understand the genotype-phenotype relationships. In this review, we describe the reconstruction of the human metabolic network, introduce the constraint based modeling approach to analyze metabolic networks, and discuss systems biology applications to study human physiology and pathology. We highlight the challenges and opportunities in network reconstruction and systems modeling of the human metabolic system.

## 1. Introduction

Metabolism is crucial to cell growth and proliferation. Deficiencies or alterations in metabolic functions are known to be involved in many human diseases. For example, the pathogenesis of diabetes results from malfunction in the regulation of metabolic pathways, leading to alterations in insulin signaling, oxidative metabolism, and lipid/fatty acid metabolism [1]. Dysregulation of the metabolic system is also implicated in carcinogenesis [2]. Most cancer cells have higher glycolytic rates, the so-called “Warburg effect” [3,4,5]. A recent study of breast cancer further uncovered alterations in glucose metabolism mediated by phosphoglycerate dehydrogenase (PHGDH) enzyme [6], whose expression was found to be associated with poor prognosis [7]. Since metabolism plays an essential role in cell growth and proliferation, genes regulating metabolism have been used as drug targets in the treatment of cancer [8,9] and other diseases involving metabolic disorders [10,11], including diabetes, atherosclerosis and fatty liver disease. Thus, understanding the human metabolic system is important for the study and treatment of complex human diseases.

As a highly-connected complex reaction network wherein the functionality and the connectivity is strongly associated [12], changes in one gene or pathway of the metabolic system could have global effects, thus systematic modeling is required to better study the function and regulation of human metabolism. Current reconstructions of the global human metabolic network provide a computational platform to integrate knowledge gained over the past 50 years of research on human metabolism [13], but more importantly, they enable a systems biology approach to study *in silico* the global effect of perturbations on the network to generate hypotheses and help understand the mechanisms underlying the genotype-phenotype relationship.

In this review, we first describe the reconstructions of global human metabolic network, and then introduce the constraint based modeling approach to analyze metabolic networks. We further discuss systems biology applications of the metabolic networks to study human physiology and pathology. Finally we highlight the challenges and opportunities in network reconstruction and systems modeling of the human metabolic system.

## 2. Reconstruction of Global Human Metabolic Network

The global human metabolic network has been manually curated based on an extensive collection and evaluation of the genomic and bibliomic data. The first two installation of the network were released in 2007: The Edinburgh Human Metabolic Network [14] and the human Recon 1 [13], each contains a list of human reactions, metabolites and gene-protein-reaction relationships. The Gene-Protein-reaction (GPR) represents functional relationships between genes/proteins (e.g., enzymes) and the corresponding reactions they catalyze or control. For example, in the human Recon 1, the genes are first mapped to their transcripts, accounting for alternative splicing. Then, based on Boolean rules of OR and AND, the transcripts are mapped to the proteins. The proteins are then mapped to reactions by Boolean rules based on the current knowledge of their effects on the reactions.

The two networks (Edinburgh Human Metabolic Network and the human Recon 1), developed independently by different research groups, consist of many different genes and reactions. The Edinburgh Human Metabolic Network contains more genes and metabolites, but was not compartmented in its initial release. Compartmentalization requires assignments of metabolic reactions into different cellular organelles (cytoplasm, nucleus, endoplasmic reticulum, mitochondria, lysosome, peroxisome, and Golgi apparatus) and accounts for the transportation and exchange of metabolites between organelles. Human Recon 1 is a compartmented network which could be used in reconstructing predictive models for systems biology studies, therefore, most of the recent applications have been based on Recon 1. An overview of the publications thus far that used Recon 1 is reviewed by Bordbar and Palsson [15]. Notably, in 2010, the compartmentalization of the Edinburgh Human Metabolic Network was completed and its current release is a compartmented, and more complete human metabolic network [16].

The reconstruction of the global human metabolic network uses a bottom-up approach. Researchers begin by compiling reactions of cellular metabolism to build a network through the collection of gene annotations, enzymes and pathway information from genome (e.g., NCBI, Ensembl) and pathway (e.g., KEGG, ExPASy) databases. Researchers then refine the network by manually collating literature evidences, including journal articles, reviews and textbooks on metabolic functions, biomass composition, growth conditions and gene-reaction associations. The constructed draft network is converted to biochemical models to evaluate the basic functionality, and simulations are performed to check for consistency with the current knowledge. The whole process runs iteratively to incorporate as much information and minimize gaps and inconsistencies. The protocol for the reconstruction process is available in [17].

The major difference in a metabolic network as compared with other biological network, e.g., Protein-Protein Interaction, Protein-DNA network, is that the metabolic network represents a biochemical system that is charge-balanced, mass-balanced and compartmentalized. This not only provides information about whether there is an interaction, but also how it happens and what it produces as a biochemical reaction, and thus can be directly converted into mathematical equations based on the biochemical reactions, for model predictions. 

## 3. Modeling and Simulation Based on Human Metabolic Network

A reconstructed human metabolic network can be represented by a system of stoichiometric reactions. This system of reactions can be modeled as ordinary differential equations, however the reaction rate constants and metabolite concentrations are typically difficult to obtain, thereby limiting their applicability to small well-studied networks. However, since the stoichiometry of metabolic reactions are not organism or context-dependent but is fixed by mass balance, one could apply Constraint Based Modeling (e.g., Flux Balance Analysis, FBA [18]) to simulate the state of the system without detailed kinetic data, assuming that the flux distributions based on the stoichiometric mass balance are at steady state or pseudo-steady state. 

Mathematical representation of reaction network and Constraint Based Modeling:

- Reactions: *S* (Stoichiometric Matrix), with *m* compounds (rows) and *n* reactions (columns). The stoichiometric coefficients are negative for the substrates of each reaction, and positive for the products.
- Flows: *v* (*n* by *1* vector) on all reactions
- Concentrations: *X* (*m* by *1* vector) of all compounds

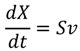
(1)

Assuming pseudo-steady state, the time derivative is zero, therefore:

*Sv* = 0 (2)

So the flux distribution *v* that satisfies this equation is in the null space of *S*.

In the human metabolic network, *n > m* results in an under-determined system that does not have a unique solution. Adding constraints permit a “feasible” solution to the system of equations, for example, a “flux capacity” constraint determines the upper and lower bounds of the flux through a reaction. Imposing mass balance and capacity constraints will define the space of feasible steady-state flux distributions of the network. Geometrically the space looks like a “flux cone” in the null space of *S*. A visualization of the “flux cone” is shown in [19] (Figure 1 in [19]) to show how the solution space could be narrowed by the steady-state and capacity constraints. Further, in FBA we define an objective function *Z*, which is a linear function of fluxes. An objective function could be

*Z* = *C^T^ V* (3)

where *c* is a column vector which assigns weights to each reaction, *c^T^* is the transpose of the vector *c*, and *V* is the flux vector through all the reactions. Optimization of the objective function *Z* identifies a unique (or multiple) set of flux configurations within the flux cone. The constrained linear optimization problem can be solved by linear programming [19,20].

The form of the objective functions, constraints and the optimization problems can vary depending on the biological applications, which are different variants of the Constraint Based Modeling. For example, the Flux Sensitivity Analysis (FSA) estimates the objective flux change in response to perturbations in some reactions of interest [21]. The Flux Variability Analysis (FVA) explores the solution space to exam the maximum/minimum fluxes for each reaction [22]. Further, current approaches to reconstruct context dependent metabolic networks are essentially different variants of the Constraint Based Modeling. A detailed review of the algorithms in the Constraint Based Modeling is provided in [23].

## 4. Systems Biology Applications of Human Metabolic Network

**Figure 1:**
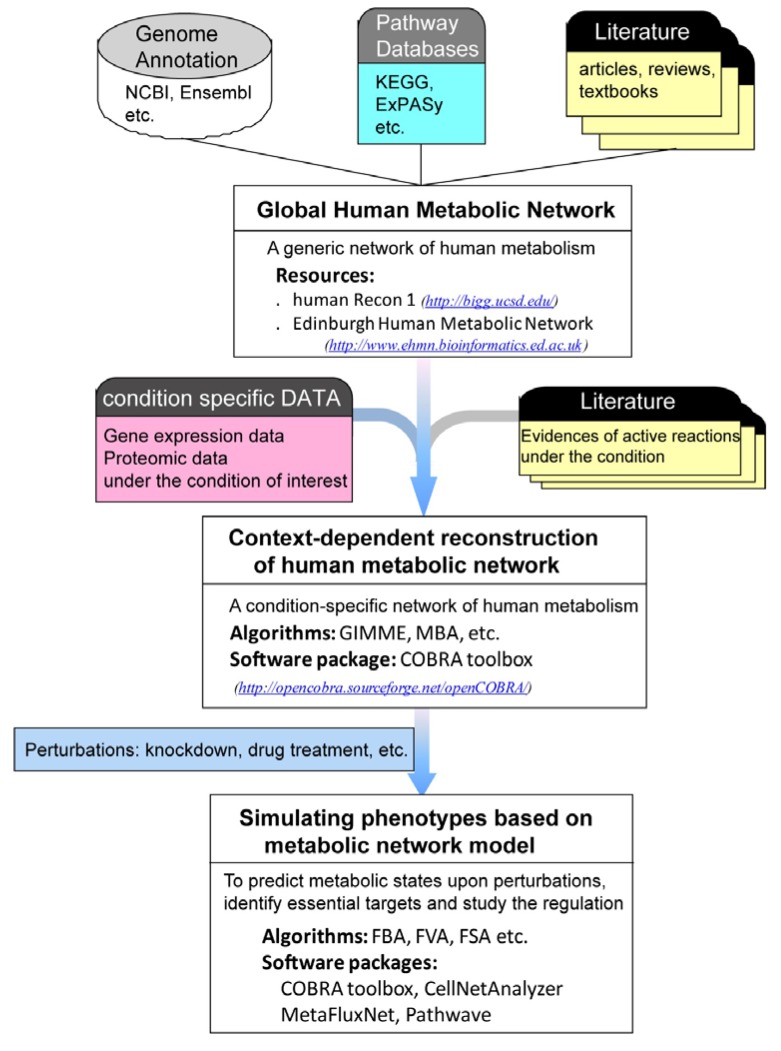
A pipeline for systems biology applications of human metabolic network. The global human metabolic network integrates literature and genomic information (including gene annotations, reactions and pathways) to provide a platform for systematic analysis and modeling. Condition-specific omics data and literature information can be incorporated into a platform to reconstruct context-dependent metabolic networks. Modeling and simulation based on context-dependent metabolic network can then be used to predict metabolic states under various perturbations, help identify gene targets and study regulation of the human metabolic system. Softwares listed in the figure: COBRA [24]: The COnstraints Based Reconstruction and Analysis toolbox. CellNetAnalyzer [25]: structural and functional analysis of biochemical networks. MetaFluxNet [26]: Analysis of metabolic fluxes in an interactive and customized way. Pathwave [27]: Identification of differentially regulated enzymes in metabolic system.

A central goal in the application of systems biology on the human metabolic network is to reconstruct and simulate context-dependent (*i.e.*, condition/cell-type/tissue/organ specific) human metabolic systems in order to generate biological hypotheses and study the physiology or pathology of cellular processes. Since the global human metabolic networks (the human Recon 1 and Edinburgh Human Metabolic Network) are generic metabolic networks that collate information from all types of human cells, the reconstruction of a context-dependent network is required prior to *in silico* analysis of the particular system under investigation. Once the context-dependent reconstruction is obtained, one can simulate the metabolic phenotypes under different perturbations to identify essential gene targets or pathways, or predict cellular responses to different treatments. A summary of the pipeline for the systems biology applications of human metabolic network is shown in Figure 1.

### 4.1. Reconstructing Context-Dependent Metabolic Network

Similar to the reconstruction of the global human metabolic networks, one could follow the protocol [17] to collate literature evidences and gene annotations, and manually identify the context-dependent reactions to reconstruct a ***condition-specific*** metabolic network. This has been applied in [28,29] to achieve comprehensive reconstructions of hepatic and neuronal cells. However, the process requires manual evaluation of thousands of papers, and curates thousands of genes and metabolites, which is lengthy, and requires tremendous effort and labor. Therefore, many studies have focused on automating the reconstruction of a cell-type or tissue-specific metabolic network, by incorporating high throughput gene expression data rather than manual curation of the cell/tissue-specific network.

Assuming changes in gene expression drives changes in the metabolic states; the basic idea in automated reconstruction is to identify active reactions by incorporating condition-specific gene expression profiles. Most reconstruction approaches start by analyzing the gene expression data to determine if a gene is “present” (highly expressed) or “absent” (low expression level) for the condition being investigated, then selects the active reactions according to their corresponding gene/enzymes’ expression level. For example, the Gene Inactivity Moderated by Metabolism and Expression (GIMME) algorithm, developed by Becker *et al.* [30], uses gene expression data to determine active and suppressed genes with an expression threshold, and determines active reactions based on the state (active or suppressed) of the corresponding enzymes. “Inactive reactions” are removed unless they are required for a desired functionality (according to a predefined objective function). Another approach to study human tissue-specific metabolic states was developed by Shlomi *et al.* [31]. The approach does not require an objective function but matches active reactions with expression data by solving a network flux to maximize the number of enzymes that are highly expressed and catalyze flux-carrying reactions. For a better quality reconstruction, Jerby *et al.* [32] developed a Model Building Algorithm (MBA), which determines the active “core reactions” with multiple sources of information including literature, transcriptome and proteomic data. The MBA then reconstructs a consistent network (no gaps or zero-flux reactions) with all the pre-defined core reactions (evidences obtained from both literature and data), adding as many of the likely active reactions (evidences obtained only from high throughput data), and as few of the other reactions. Nevertheless, manual curation is necessary with this approach to collate and analyze the literature and high-throughput data.

### 4.2. Simulating Phenotypes Based on Metabolic Network Model

Constraint based modeling and simulation based on a condition-specific human metabolic network can be used to predict the flux distribution in the network for that specific metabolic phenotype. Such *in silico* analysis can be used to generate hypotheses on the cell growth, ATP production, or the states of specific metabolic functions upon perturbation [33].

Modeling and simulations of a metabolic model of human kidney reconstructed with the GIMME algorithm were used to evaluate the metabolic phenotypes associated with the side-effects of a drug treatment [34]. The side effect of a particular drug is determined by its off-targets, which are the enzymes/genes that are not the therapeutic targets but nevertheless are predicted to bind and be inhibited by the drug. FBA was performed on the perturbed network where the reactions catalyzed by the off-target enzymes were inhibited by the drug, to evaluate the systematic consequences of the drug and determine if the treatment leads to deficiency in metabolic functions [34]. This systematic approach has also been applied to metabolic disorders of the liver [32] and cancer cells [35,36], and was able to correctly identify many of the genes essential for the metabolic disorders [32], interpret metabolic state-changes (e.g., Warburg effects [36]), and predict drug targets for the metabolic system [35]. In the cancer study [35], gene expressions in the cancer cell lines are analyzed to identify the highly expressed metabolic enzyme-encoding genes in the cancer, which are used on the MBA algorithm to reconstruct a “cancer metabolic network model”. FBA is then applied to predict metabolic states (cell proliferation) across different gene knock-downs. Specifically, in each prediction one could turn off the reaction associated with the gene that is knocked-down and apply FBA on the constrained model to determine if cell proliferation (represented by an objective function) is reduced. The genes predicted to be important for cancer metabolism are confirmed to be highly essential in a shRNA gene knockdown dataset which lists experimentally identified cancer growth-supporting genes. FBA is also applied on non-cancer cells (reconstructed metabolic network with expression data of normal cells) to determine genes important for metabolism of normal cells. Genes that only affect cancer cells are predicted to be drug targets. Many known targets of FDA-approved metabolic anticancer drugs have been re-discovered with this approach. Folger *et al.* [35] further simulated double gene knockdowns to explore combinations of synthetic lethal drug targets.

In summary, the reconstruction and simulation of human metabolic networks enable a systematic study of the regulatory system and is applicable to a wide variety of cases. One could rely then on *in silico* perturbations to predict novel targets, interpret systemic effects, and identify alterations in the metabolic states to better understand the genotype-phenotype relationships. 

## 5. Challenges and Perspectives

### 5.1. The Consolidation of the Human Metabolic Network

Current reconstructions of the global human metabolic network are incomplete due to limitations in our knowledge of the complexity of the human genome. Both the human Recon 1 and Edinburgh Human Metabolic Network have gaps and missing parts. For example, there are many reactions in the networks that do not have corresponding regulatory genes/enzymes annotated. For the reactions with known genes/enzymes in either network, there are only around one thousand genes that are common to both, with some collated in only one of the networks. It would be better to combine the two reconstructions to achieve a more comprehensive network. Nevertheless, the integration would be difficult since the standards are different between the two networks—Different compound names, different reaction names, and different IDs are used for the network components.

The community needs to drive the effort for better reconstruction and integration of these network models. A community approach of “reconstruction jamborees” [37,38] was suggested for modeling the metabolic network of different species and systems. A “jamboree” is a focused work meeting that promotes collaborations between experts from diverse fields (e.g., genetics, physiology, metabolomics, engineers, and computer science), to define and evaluate protocols, standards and ontologies for the curation of models, and to resolve discrepancies. Such a collaborative effort would help refine and update the human metabolic network to further enhance the applications of these networks. 

### 5.2. Novel Approaches to Reconstructing Context-Dependent Networks

Applications of the metabolic model require reconstruction of context-dependent networks, thus computational approaches to automate the generation of condition-specific models are of great interest. Current approaches rely on present and absent (high/low expression) of the genes to determine potentially active reactions, which could be problematic since a small change in the threshold in the expression analysis could result in very different lists of present and absent genes and affect the reconstruction. Most genes’ expressions are not naturally bimodal but have many levels. A discretization process separates such expression levels into ON and OFF states with some arbitrary thresholds (e.g., based on percentile, clustering, or differential expression), thereby the analysis based on discretization will be affected by the threshold that is chosen. A change in the threshold will change many of the genes’ ON/OFF categorization, which could affect the network reconstruction, since the reconstruction process is also bimodal with reactions being absent or present according to the ON and OFF state of the genes. For example, a gene expressed a little lower than the median may be classified as absent/OFF, then the reaction it is associated with may be removed. However a gene is not necessary in an entirely OFF state when expressed at a low level, and a reaction may not necessary be completely abandoned just because there are fewer enzymes than normal. Current approaches rely on the stoichiometric constraints to mitigate the possible inconsistencies. Future reconstruction approaches could impose more realistic models of the functional relationship between genes and reactions. For example, a novel approach developed by Colijn *et al.* [39] associates gene expression levels with the constraints on the reactions, *i.e.*, the lower a gene is expressed, the lower the flux that could be conducted through its corresponding reactions. Although it could be more sensitive to changes (noises) in the gene expression by directly associating the gene expression level with flux bounds, the approach is less dependent on the discretization of the gene expression and the present/absent call of the reactions, which could provide a more realistic model. It has been applied to bacteria to predict metabolic modulators and responses to different drugs [39].

With the rapid development of metabolomics, metabolic flux data becomes more readily available and novel approaches are being developed to more efficiently monitor flux distribution in mammalian cells with Metabolic Flux Analysis, by employing isotope-labeling (e.g., C13 label) techniques [40]. Researchers have been studying metabolic alterations in human cells by comparing gene expression profiles with the associated metabolic fluxes measured with isotope-labeling [6,41]. Future modeling and reconstruction of metabolic networks could also incorporate metabolic flux data to determine condition-specific flux distributions and metabolic states.

### 5.3. Reconstruction of Multi-Cellular Metabolic System

Context-dependent metabolic models of different cells could be incorporated to achieve higher level systems to study cell-cell interactions. A recent study by Bordbar *et al.* [42] incorporated metabolic models of human alveolar macrophage and *M. tuberculosis* to study host-pathogen interactions. Simulation of this metabolic system under different infection states helped identify differentially active pathways and potential gene targets. Another pioneering work by Lewis *et al.* [29] reconstructed a brain metabolic network for different types of neurons and their interactions with astrocytes, to study energy metabolism in the different cell types. The multi-cellular *in silico* model was used to analyze the gene expression data to identify changes in the metabolic states as well as important genes in Alzheimer’s disease. In both studies, the incorporation of the metabolic networks was based on manual curation, since there are no computational approaches for reconstructing a multi-cellular metabolic system to date. In the future we should expect novel approaches and applications to higher level systems modeling that involve different cells and tissues.

### 5.4. Incorporating Regulations on Multiple Layers

The gene-reaction associations in current reconstructions of the human metabolic networks account for enzymes, isozymes, transcript variants and protein complexes, but however are limited to metabolic-related genes and enzymes. There are other layers of regulation of gene expression and enzyme activity, including transcriptional regulation, post-transcriptional and post-translational processes. Many genes involved in transcriptional or post-transcriptional regulation of the metabolic genes or enzymes could also affect the metabolic states, which are excluded from current applications. Although it is difficult to have a comprehensive model due to limitations of our knowledge of biological regulation, we could incorporate more information into the metabolic network to provide a better model of the regulation of the human metabolic system. Given the increasing amount of transcriptome data, protein interaction data and other high-throughput data, they could be capitalized upon to enhance the regulatory model. Future studies should consider incorporating multiple layers of regulation to better understand the molecular mechanisms behind the regulation of human metabolism.
